# Morphological adaptation of the calamistrum to the cribellate spinning process in Deinopoidae (Uloboridae, Deinopidae)

**DOI:** 10.1098/rsos.150617

**Published:** 2016-02-24

**Authors:** Anna-Christin Joel, Ingo Scholz, Linda Orth, Peter Kappel, Werner Baumgartner

**Affiliations:** 1 RWTH Aachen University, Institute of Biology II, Worringerweg 3, Aachen, Germany; 2 JKU Linz, Institute of Biomedical Mechatronics, Altenberger Straße 69, Linz, Austria

**Keywords:** *Zosis geniculata*, *Deinopis subrufa*, cribellar, fibre processing, fibre extraction, morphological adaptation

## Abstract

Spiders are famous for their silk with fascinating mechanical properties. However, some can further produce, process and handle nano fibres, which are used as capture threads. These ‘cribellate spiders’ bear a specialized setae comb on their metatarsus (calamistrum), which modifies cribellate nano fibres to assemble a puffy structure within the capture thread. Among different species, the calamistrum morphology can differ remarkably. Although a model of thread production has been established for *Uloborus plumipes*, it is not resolved if/how different shaped calamistra influence the production process. We were able to transfer the model without restrictions to spiders with different shaped calamistra. Fibres are not locked between setae but are passing across a rather smooth surface-like area on the calamistrum. This area can be relocated, explaining the first morphological difference between calamistra, without changing the influence of the calamistrum on fibres. By performing an elongated leg movement, contact between fibres and calamistrum could be adjusted after finishing thread production. This movement has to bring the thread in contact with the second morphological peculiarity: cribellate teeth. We suggest these teeth are used to handle the thread independently of the spinnerets, a feature only necessary for spiders, which do not move during web construction.

## 1. Introduction

**Figure 1.**
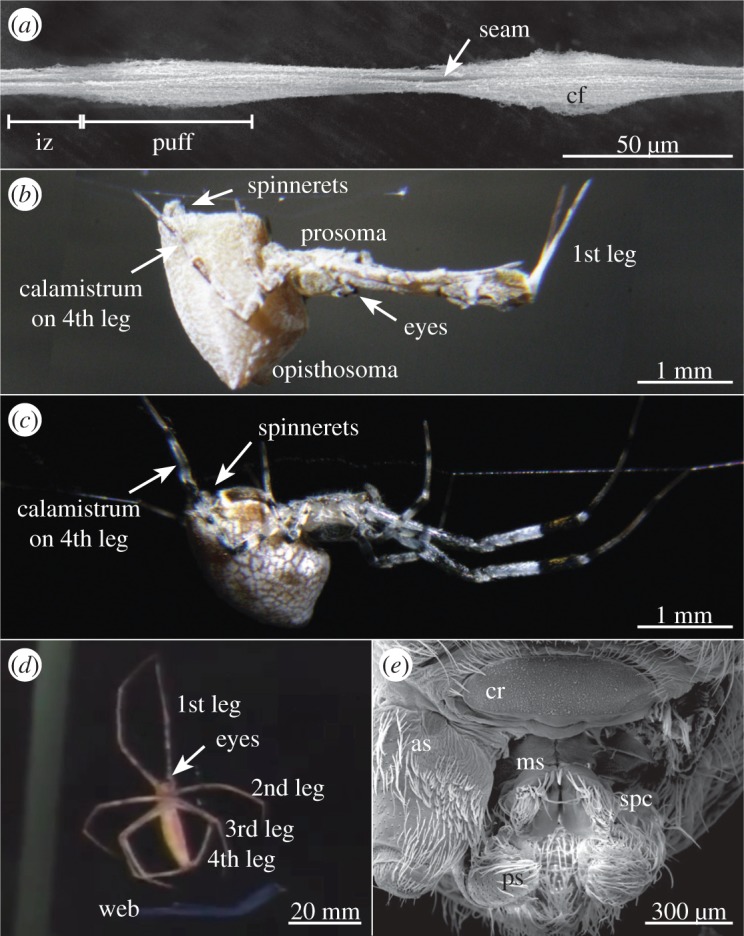
Different cribellate spiders and a typical capture thread. (*a*) Cribellate capture thread of *Zosis geniculata* with the typical puffy structure consisting of cribellate fibres (cf). iz, intermediate zone. (*b*) Female *Uloborus plumipes* in its resting position during the day. (*c*) Female *Z. geniculata* in its resting position during the day. (*d*) *Deinopis*sp. during capture thread production. This picture is extracted from a youtube-video (see Material and methods). The scale bar is therefore estimated based on the mean body size of *Deinopis subrufa*. (*e*) Overview of the spinnerets of *Z. geniculata* showing the typically pairwise arrangement of the spinnerets. Only the cribellum (cr), where the cribellate fibres emerge, is not paired in this species. By removing one anterior spinneret (as), the median spinnerets (ms) bearing the spigots of the paracribellum (spc) become visible. They produce the paracribellate substructure. The axial fibres emerge from the spigots of the glandulae pseudoflagelliformes on the posterior spinnerets (ps). (*a*,*e*): scanning electron microscope (SEM) images.

Spiders are well known for their ability to produce silk with special mechanical properties [1–3]. However, not only the mechanical properties of their silk are fascinating, but some spiders have also evolved a way to produce, process and handle nano fibres. These spiders belong to the cribellate spiders, which use, in contrast to ecribellate spiders, a capture wool instead of glue droplets to capture prey [4–6]. The capture wool consists of nano fibres called cribellate fibres, which are organized as a mat surrounding two larger fibres (axial fibres; figure 1*a*). In *Uloborus plumipes* (figure 1*b*) the cribellate mat is fixed at the axial fibres in the intermediate zones, most probably by a substructure consisting of paracribellate fibres [7]. The cribellate fibres shape the characteristic puffy structure of a cribellate thread by an irregular alignment of fibres within puffs and a more regular alignment in the intermediate zones, connecting two puffs [7–11]. Processing of cribellate fibres is performed by comb-like row of twisted and curved setae on the metatarsus of both fourth legs called calamistrum [7].

**Figure 2.**
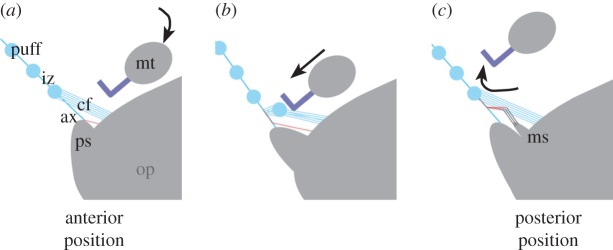
Lateral overview of the model for the cribellate spinning process. During the production of the cribellate thread, the metatarsus (mt) bearing the calamistrum moves in an oval movement over the spinnerets (arrows), coming in contact with the cribellate fibres (cf) during the posterior movement (combing movement; *a* to *b*). Owing to this, cribellate fibres are processed and a puff is formed (*b*). During the anterior movement (*c*) of the metatarsus, it is lifted and contact between calamistrum and fibres inhibited. Hence, an intermediate zone (iz) is produced. The median spinnerets (ms) show a spreading, extending their elongated spigots of the paracribellum in the extracted cribellate mat. This infiltration with paracribellate fibres (in hatched red) and the synchronously adducting posterior spinnerets (ps) enable the production of linkages in the intermediate zones, finally connecting cribellate mat and axial fibres (ax). op, opisthosoma. Scheme made following the description of Joel *et al*. [7] and the observation of species in this study.

The cribellate fibres are extracted from the cribellum, an unpaired spinning plate anterior to the other spinnerets (figure 1*e*) [12]. With about 5000 spigots they outnumber the elongated spigots of the paracribellum on the median spinnerets (synthesizing the paracribellate fibres), as well as the spigot of the glandulae pseudoflagelliformes (synthesizing one axial fibre) situated on each posterior spinneret [9,13–15]. All these three involved spinnerets as well as both fourth legs bearing the calamistrum show a coordinated movement during capture thread production, which are incorporated in a model of the cribellate spinning process of *U. plumipes* [7]:

Both fourth legs form a unit anterior to the spinnerets with one being the combing leg, placing its tarsus on the metatarsus of the other fourth leg, called the supporting leg [16,17]. The calamistrum of the combing leg is lowered in an oval movement while moving from the anterior of the spinnerets to posterior with a repeat rate of 8–10 Hz in *U. plumipes* [7,8]. During this movement (‘combing movement’), the calamistrum gets in contact with the cribellate fibres and processes the cribellate fibres to form one puff per stroke (figure 2*b*). The posterior spinnerets move synchronously to the movement of the fourth legs, abducting during the combing movement [7,11]. During the faster retraction of the combing leg in the anterior direction (‘non-combing movement’), the median spinnerets spread apart, probably infiltrating the cribellate mat (figure 2*c*). When retracting again they fix the cribellate mat to the axial fibres (figure 2*a*), thereby producing the linkage visible in the intermediate zones.

Up to now, the here presented model about cribellate capture thread production only includes observations on *U. plumipes* [7]. Such a single species observation does neither allow any validation of the model nor any abstraction or estimation about possible factors involved in handling or processing the nano fibres. This is unfavourable as it is to date not determined, precisely how the calamistrum process the cribellate fibres. Nevertheless, it is well known that the calamistra can differ remarkably, e.g. by having one or several rows of setae or by bearing or not bearing a teeth-like substructure [18,19]. The aim of our work is therefore to analyse the morphological and behavioural differences or similarities during capture thread production and evaluate their relation towards each other as well as their impact on the model. For the validation of the model we have chosen two close relatives to *U. plumipes* with similar spinneret morphology [20]. One is likewise a solitary living orb-weaving spider belonging to the Uloboridae: *Zosis geniculata* (figure 1*c*). As Deinopidae, the second spider chosen belongs to the same superfamily (Deinopoidae) (*Deinopis*sp., figure 1*d*) [21,22]. Deinopidae are net-casting spiders, catching their prey by throwing themselves and their web onto approaching prey [23,24]. Their web building behaviour differs from typical orb-weaving spiders by, for example, keeping their body on spot during web construction [23]. Furthermore for the Deinopidae *Menneus*sp., the occurrence of teeth on the calamistrum is described [18]. Such teeth are missing in Uloboridae. Differences in the capture thread production therefore can reflect differences in the calamistra morphology and/or different needs during web construction itself, whereas similarities hint to more preserved features of the cribellate spinning process.

## 2. Material and methods

### 2.1 Study animals

Adult female specimens of the spiders *U. plumipes* (Lucas, 1846) and *Z. geniculata* (Olivier, 1789) were taken from our laboratory colony and raised separately in Petri dishes (Ø 9 cm) with roughened surfaces as web support. For easier observation of the web building behaviour during the night, their diurnal rhythm was shifted 12 h: white light was provided from 18.00 to 07.00. For observation, spiders were illuminated from 07.00 to 18.00 using red LEDs (Paulmann Licht GmbH, Springe, Germany). Once or twice a week, spiders were fed with one juvenile cricket (*Acheta domestica*) or one to two fruit flies (*Drosophila melanogaster*). Once per month water was provided by sprinkling their webs.

Seventy per cent ethanol preserved specimens of *Deinopis subrufa* (L. Koch, 1879) of undefined age were kindly provided by the Australian Museum (Sydney, Australia) and the Queensland Museum (Brisbane, Australia).

### 2.2 Analysis of the cribellate spinning process

For analysing the cribellate spinning process, video recordings of *U. plumipes*and *Z. geniculata* were performed. Spiders were transferred into modified Petri dishes with dark adhesive tape as background and web support. One side was cut open at about 1/4 of the Petri dish diameter and sealed again with a microscope slide for undisturbed observation. Recordings were performed under red light. Spiders were observed from top and side view using two binocular microscopes with 10–40× magnification. As the spider hangs upside down in its webs during thread production (figure 1*b*,*c*), the top view resulted in an observation of the ventral side of the spider. The microscopes could be moved almost vibration-free due to Teflon feet. A digital reflex camera (EOS 550D; Canon, Tokyo, Japan) was attached to both binocular microscopes. Recording speed was 50 fps. Online available video recordings of *Deinopis*sp. were used according to Nelson & Fijn [25] (table 1). Special care was taken for determining the frequency by only evaluating recordings where speed manipulation can be excluded.

**Table 1.** Links to the clips used for analysing the cribellate silk production in *Deinopis* sp.

link	date accessed	spider	special data collected
https://www.youtube.com/watch? v = GWY5cBueiHk	13 Aug 2015	*D. subrufa*	angle between body and thread (*α*), elongated leg movement, figure 5*b*
https://www.youtube.com/watch? v = _NthIs56OkY	8 June 2015	*Deinopis* sp.	angle between body and thread (*α*), frequency
https://www.youtube.com/watch? v = 1H4ayAN1u7o	8 June 2015	*Deinopis* sp.	frequency
https://www.youtube.com/watch? v = e4MhaEgrDV4	13 Aug 2015	*Deinopis* sp.	none
https://www.youtube.com/watch? v = gryaUTpPgGU	13 Aug 2015	*Deinopis* sp.	figure 1*c*
http://footage.framepool.com/de/bin/12250,deinopidae,spinnennetz,australien/	10 June 2015	Deinopidae	no. 640–469–842 and 809–396–662: elongated leg movement

Videos were analysed using Keyence VW-9000 Motion Analyser (v. 1.4.0.0; Keyence Cooperation, Osaka, Japan) or a self-written script for point tracking after fractionizing the recording in single frames (Matlab R2014b v. 8.0.0.783, The MathWorks Inc., Natick, USA).

### 2.3 Calamistrum characterization

Seventy per cent ethanol preserved spiders of *U. plumipes*, *Z. geniculata* and *D. subrufa* were gradually transferred to 100% ethanol and dried using hexamethyldisilazane (Merck, Darmstadt, Germany). Specimens were sputter coated with a 10 nm gold layer (Hummer; Technics Inc., Alexandria, USA) before examination in a scanning electron microscope (SEM: 525 M; Philips AG, Amsterdam, The Netherlands).

### 2.4 Fixation during silk production

Spiders (*U. plumipes*) were transferred into Petri dishes with roughened surfaces. They were shock frozen with cold spray (TC KC400C; Toolcraft, Hirschau, Germany) during production of cribellate threads, immediately transferred to −20°C and stored for two weeks on silica gel desiccant. After another two weeks at +4°C, they were transferred to room temperature. Specimens were sputter coated and examined using an SEM.

### 2.5 Conglutination of the setae of the calamistrum

Adult female spiders of *U. plumipes* were anaesthetized with diethyl ether and fixed with needles onto a Petri dish. With the help of a needle, a small droplet of superglue (Uhu Sekundenkleber blitzschnell Supergel; Uhu GmbH & Co. KG, Brühl, Germany) was picked up and brought near to the gap between calamistrum and the second row of setae nearby. The glue was sucked up by this gap, gluing the setae of the calamistrum together. For observation of the capture threads produced by such a modified spider, samples of freshly produced threads were taken and examined using an SEM. As a control of successful modification, the spiders were frozen at −20°C and dried for a week at room temperature using silica gel desiccant. Specimens were sputter coated before examination in an SEM.

## 3. Results

### 3.1 Differences in the morphology of the calamistrum

**Figure 3.**
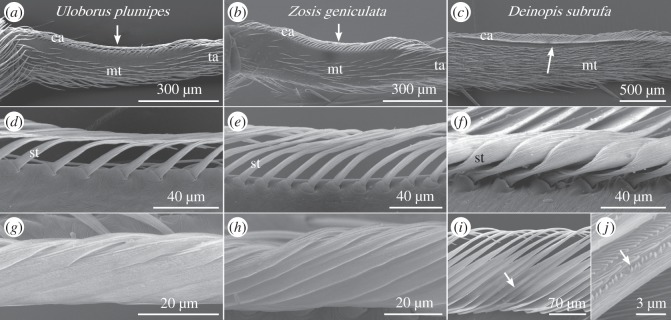
Morphological differences between the calamistra of Uloboridae and Deinopidae. (*a*–*c*) Overview of the calamistra (ca) on the metatarsus (mt) of *U. plumipes* (*a*), *Z. geniculata* (*b*) and *D. subrufa* (*c*). The arrow points towards the surface-like area on the calamistrum, observed in all three species. ta, tarsus. (*d*–*f*) Detail of the calamistrum, showing the setae (st) emerging from the cuticle. In *U. plumipes* (*d*) as well as *Z. geniculata* (*e*), large gaps between the setae are observable. By contrast, the setae of the calamistrum of *D. subrufa* overlap each other, building a surface-like area before bending (*f*). (*g*–*j*) Detail of the calamistrum showing the tip of the setae orientated parallel to the roots of the setae. The tips of *U. plumipes* (*g*) and *Z. geniculata* (*h*) are forming a surface-like area though overlapping setae. The tips of *D. subrufa* are not building a surface-like area but show wide gaps between the single setae (*i*). On the edge of each seta (indicated by an arrow), one row of irregular teeth could be observed (*j*). SEM images.

Following the literature about the similar web building behaviour, one would suggest a morphologic similar calamistrum in both Uloboridae as well. Indeed, the calamistra of *U. plumipes* and *Z. geniculata* showed striking similarities regarding their morphology (figure 3). At the root, single setae were easily distinguishable, forming one row with gaps between the setae (figure 3*d*,*e*). Tracing a setae to its tip, the setae bends about 90° while enlarging and flattening its breadth (figure 3*g*,*h*). This led to single setae overlapping the adjacent one. There were no more gaps visible between two setae, thereby simulating a surface-like area parallel to the roots of the setae within the calamistrum. The setae were completely covered with grooves and teeth were not visible in either species.

By contrast, the calamistra of *D. subrufa* had a completely different shape. Directly after emerging from the cuticle surface, the setae flattened and enlarged their breadth. This led to a covering of the adjacent setae and therefore a formation of a surface-like area, this time orthogonal to the roots of the setae (figure 3*f*). The setae also bend about 90° and reduce their breadth again, leading to large spaces between the setae tips in a parallel plane to the roots (figure 3*i*). The complete setae were covered with grooves. Furthermore, the thinner tips of the setae bore one irregular row of cribellate teeth (figure 3*j*).

As the calamistrum is required to process cribellate fibres, such striking morphological differences in the shape and structuring of the calamistra should result in differences within the cribellate spinning process. Hence, we compared the model for cribellate thread production with our observations of *Z. geniculata* and *Deinopis*sp. to unite functional morphology and the spinning process.

### 3.2 The cribellate spinning process

Analysing thread structures revealed the same basic principles as observed in *U. plumipes*: a puffy structure (figure 1*d*) with an obvious seam, demonstrating cribellate fibres were organized as a mat surrounding the paired axial fibres. Furthermore, the capture threads of *Z. geniculata* showed interconnections between cribellate mat and axial fibres.

With a frequency of 8.68±0.40 Hz (*n*=3) and 8.44±1.12 Hz (*n*=2) for *Z. geniculata* and *Deinopis* sp., respectively, both spider species moved their combing leg in an oval manner over their spinnerets producing one puffy structure per stroke (*Z. geniculata*). Starting at the anterior position, combing took place during the posterior movement of the combing leg indicated by a lowering of the metatarsus. Furthermore, the posterior spinnerets abducted simultaneously, reflecting the behaviour observed in *U. plumipes*. When reaching the posterior position, the leg’s velocity increased about three times while retracting the leg to the anterior position. During this retraction (non-combing movement), the median spinnerets of *Deinopis* sp. and *Z. geniculata* spread apart. Thread production stopped always with the combing leg at the posterior position.

Our observations show the main components of the cribellate spinning process in *U. plumipes* are conserved, permitting us to conclude that the model of the cribellate spinning process can be transferred without restrictions to our investigated species.

### 3.3 Characterizing the contact between cribellate fibres and calamistrum

**Figure 4.**
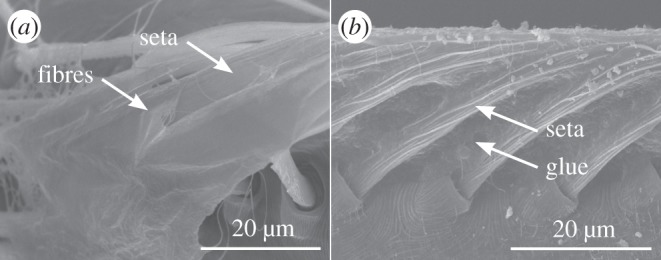
Cribellate fibres are passing across the calamistrum. (*a*) Shock-freezing experiments performed with *U. plumipes* showed a mat of fibres covering the surface-like area near the tip of the calamistrum. No fibres or residuals of fibres were observed passing between two setae. (*b*) Conglutinated calamistrum, inhibiting a passing of fibres between the setae. SEM images.

The established model of the cribellate spinning process however does not include the characterization of contact between calamistrum and cribellate fibres. In order to do so, one has to determine whether cribellate fibres are actually trapped between the setae of the calamistrum as suggested by Peters [11] or whether the fibres are passing across the calamistrum. Fixing *U. plumipes* during capture thread production by shock-freezing indicated fibres are passing across the calamistrum without passing between the setae: no fibres or residuals of fibres were found between the setae but a mat of fibres covering the surface-like area of the calamistrum was often observable (figure 4*a*). We were able to support our findings, by conglutinating the setae of the calamistrum and inhibiting a passing through of fibres (figure 4*b*). Such modified spiders were still able to produce normal cribellate threads. We reasoned, that cribellate fibres are neither trapped nor passing through the calamistrum, but contact between calamistrum and cribellate fibres occurs most probably only at the surface-like area of the calamistrum.

**Figure 5.**
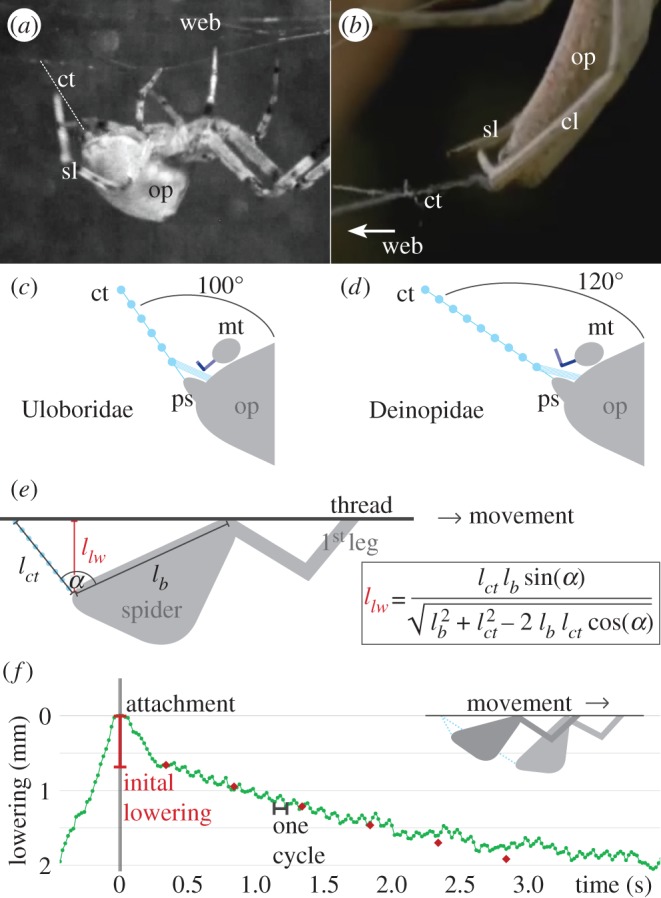
Reconstructing the contact between calamistrum and cribellate fibres. (*a*) Overview of Uloboridae *Z. geniculata* during capture thread production. For radial web construction, the spider is moving through the web. The cribellate thread (ct) is highlighted with white stripes. Note the angle is optically distorted in this picture and therefore larger than the original one. op, opisthosoma; sl, supporting leg. (*b*) Close-up of the Deinopidae *Deinopis* sp. during capture thread production. The spider does not move through the web, as the web is modified to a net-casting device (here on the right side, compare to figure 1*d*). cl, combing leg; ct, cribellate thread; op, opisthosoma; sl, supporting leg. This picture is extracted from a youtube-video (see Material and methods). (*c*,*d*) Reconstruction of the contact between calamistrum on the metatarsus (mt) and cribellate thread (ct) with an angle of 100° (*c*), respectively, 120° (*d*) between thread and opisthosoma (op). For easier comparison, (*d*) is twisted to the same orientation as (*c*). Contact is shifted towards the base of the setae by enlarging the angle. This reflects the changed position of the surface-like area (highlighted in dark). Calamistrum morphology adapted to specimen, not true to scale. ps, posterior spinnerets. (*e*) Trigonometric calculation, resolving the necessity of lowering the opisthosoma to keep the angle (*α*) between opisthosoma and cribellate thread constant despite elongation of the thread. *l*_*ct*_, length of cribellate thread; *l*_*lw*_, lowering of the opisthosoma; *l*_*b*_, length between gripping point on thread and spinnerets, simplified as the body size of the spider. (*f*) Example for *U. plumipes*’ lowering of the opisthosoma over time, equivalent to the elongation of the thread (green dotted line). The process started with an initial lowering, where no combing movement took place. Each combing cycle showed a lifting of the opisthosoma during the posterior movement of the combing leg. The red rectangles showed the calculated measurements with the formula presented in (*e*) with *l*_*b*_=5 mm (average body size of *U. plumipes*), *α*=100° and an elongation of the thread of 0.63 mm s^−1^ (average production speed in *U. plumipes*), including an initial lowering of 0.7 mm within 0.3 s after attachment.

To support this hypothesis, we reconstructed the contact between calamistrum and thread. Therefore, we measured the angle between opisthosoma and produced capture thread, called ‘*α*’ (figure 5*a*–*e*). Including the orientation of each specimen’s calamistrum towards the opisthosoma, the morphology of the different calamistra and the understanding that fibres are passing across the calamistrum, *α* can be used to recreate the contact between thread and calamistrum.

Interestingly, *α* was remarkably constant within each species: in *U. plumipes* *α* was about 100°, whereas in *Deinopis* sp. *α* was about 120°. Remodelling the contact between calamistrum and cribellate fibres, as described previously, we found an *α* of 100° leading to contact between cribellate fibres and the surface-like area near the tips of the calamistrum in *U. plumipes* (figure 5*c*). A difference of 20° however already leads to a shifted contact area between setae and cribellate fibres. A higher (respectively, more obtuse) *α* displaced the contact more towards the base of the calamistrum in *Deinopis* sp. (figure 5*d*). This result resembles the towards the base shifted surface-like area of the calamistra of *D. subrufa*.

### 3.4 Controlling the contact between fibres and calamistrum

Having an angle-sensitive area where contact between thread and calamistrum occurs, the spider needs to permanently countervail an elongating thread. Such an elongating thread would otherwise automatically lead to a stepwise increasing *α*, hence displacing the area where contact occurs. To compensate for this effect, *Z. geniculata* and *U. plumipes* showed an adapting movement of their opisthosoma during laying the capture thread spiral: after fixing the capture thread at the radial frame, they lowered their opisthosoma once initially without performing any combing movement. Afterwards an oscillating but constantly lowering of the opisthosoma took place during which one capture thread was spun between two radial threads (figure 5*f*). Trigonometric calculations revealed this lowering can compensate for the effect of the elongating thread and help keep *α* constant (figure 5*e*,*f*). The oscillation within the lowering again reflected the movement of the combing leg: the spider lifted its body slightly each time, the combing leg would otherwise lower *α* during its posterior movement. After finishing one capture thread, the opisthosoma was lifted again to fix the thread to the radial frame.

Such a lowering of the opisthosoma could not be observed in *Deinopis* sp. It kept its body on the spot during the whole thread production process and only changing the orientation of its spinneret to the left or right site. Furthermore, no initial lowering of the opisthosoma was observed and cribellate thread production started immediately after fixation at the radial frame. To keep *α* constant, *Deinopis* sp. seemed to have another strategy: gripping the web with its third legs and adjust *α* by moving the whole web. Interestingly, *Deinopis* sp. showed an 1.7±0.2 longer posterior combing movement of its fourth leg in the last cycle before fixing the finished thread to the radial frame (*n*=3). After such an elongated movement, the cribellate thread stuck to the calamistrum, in contrast to what was typically observed. This enabled the spider to move the thread independently of its spinnerets.

A summary of the results is given in table 2.

**Table 2.** Summary of the results.

	family	Uloboridae		Deinopidae
	species	*U. plumipes*	*Z. geniculata*	*Deinopis*sp.
morphology	surface-like area on calamistrum	near tip		near basis
	teeth on calamistrum	no		yes
combing process	frequency (Hz)	10.4±0.1^*a*^	8.7±0.4	8.4±1.1
		*n*=2	*n*=3	*n*=2
	relation of velocity of posterior leg	3.7±1.2×	3.1±0.9×	3.0±0.3×
	movement to anterior leg	*n*=3	*n*=3	*n*=2
	movement (times slower)			
	combing position^*b*^	type 2		
	start capture thread production	anterior position		
	stop capture thread production	posterior position		
	lowering of metatarsus	posterior leg movement		
	abduction posterior spinnerets	posterior leg movement		
	abduction median spinnerets	anterior leg movement		
behaviour	lowering of opisthosoma	yes		no
	direct start of combing after attachment	no		yes
	angle between opisthosoma and thread	100°	not determined	120°
	elongated last leg movement	no		yes
	picking up of thread	no		yes

^*a*^Data taken from Joel *et al*. [7].

^*b*^Definition after Eberhard [17].

## 4. Discussion

### 4.1 The cribellate thread production model

We found the previously proposed model of *U. plumipes*cribellate thread production also applies to the Uloboridae *Z. geniculata*and the Deinopidae *Deinopis* sp. [7]. Likewise the cribellate capture thread of *Z. geniculata*was organized as a mat, connected to the axial fibres within the intermediate zones, resembling the structure already found in *U. plumipes* [7]. Having a closer look at the literature, one also finds the seam of the cribellate mat and connections described or depicted for*D. subrufa*’s capture thread [20]. The median spinnerets of both species showed spreading during the non-combing movement, producing the connections between cribellate mat and axial fibres according to the model (figure 2). Combing took place during the posterior movement of the fourth legs indicated by a lowering of the metatarsus. Hence no retracting anterior leg movement occurred when capture thread production was interrupted.

Besides proving the model more broadly valid, we could further refine the model and exclude that cribellate fibres are trapped between the single setae in *U. plumipes* during the combing movement. It was hypothesized, that such locking of the fibres facilitates pushing the cribellate fibres together forming one puff [7,11]. It remains to be studied, if the exclusion of this hypothesis directly rules out a compression of the nano fibres. Finding such a correlation would support the common hypothesis that electrostatical charging plays an important role during thread formation [7,8,26,27].

### 4.2 Differences in calamistra morphology are accompanied by different web building behaviours

Although it still remains unclear, how the cribellate nano fibres are processed in detail, we were able to characterize where processing of cribellate fibres on the calamistrum occurs. In all observed specimens, cribellate fibres were passing over a similar structured and rather smooth surface-like area on the calamistrum. This area however was shifted from the tip of the setae as observed in *U. plumipes*and *Z. geniculata*towards the roots of the setae in *D. subrufa*. This changed the overall morphology of the calamistrum, strikingly without influencing the general properties of the cribellate spinning process. The shifted surface-like area between both families was accompanied by a changed *α* (angle between opisthosoma (reflecting the position of the calamistrum) and thread). We observed *Deinopis* sp. using their third pair of legs to move the whole web during thread production in a manner that probably maintained a constant *α* for the correct processing of fibres. This matches with an earlier description of *D. subrufa*’s web building behaviour, entitling the third legs to move constantly through the web during web production while the first and second legs have fixed places [23]. Uloboridae however lowered their opisthosoma constantly in addition to adapting their body position during each single combing cycle.

The modified morphology of the calamistrum as well as the different strategies for controlling the contact area are not restricted to our three species. Comparing the calamistra of other Deinopidae like *Menneus* sp. or *Deinopis spinosa* [18,28] with calamistra of other Uloboridae like *Miagrammopes animotus*, *Hyptiotes cavatus* or *Polenecia producta* [11,26,29,30], it can be observed that Deinopidae always have a surface-like area near the base of their setae while Uloboridae always have one near the tip. Furthermore, not only *Z. geniculata* and *U. plumipes* show an abdominal lowering, but this behaviour is also described for *Hyptiotes paradoxus* and *Uloborus walckenaerius* [31,32]. Owing to such an adaptation, spiders loose several micrometres of capture thread by having to initially lower their opisthosoma before thread production can start with the correct contact between thread and calamistrum. Peters [11] already described for *U. plumipes* that about 600 μm of the following capture thread was not covered with cribellate fibres without any further explanation. Similar observations were made for *P. producta* [33]. By moving the web/thread instead of its whole body, *Deinopis* sp. does not need such initial lowering of its body and capture thread production starts immediately after fixation. Hence the complete capture thread can be covered with cribellate fibres, enhancing the sticky area of one web [20].

We suggest these *α*-dependent adaptations of the calamistrum morphology as well as the strategies of controlling *α* are conserved within each family and probably reflect different needs during the construction of the web. These differences between Uloboridae and Deinopidae, however, do not influence the general capture thread production model, but give further insight into the importance of correct contact between cribellate fibres and calamistrum for successful processing.

### 4.3 Function of the cribellate teeth

Beside the shifted surface-like area on the calamistrum, we found a second morphological peculiarity on *D. subrufa*’s calamistrum: teeth near the tips of their setae. Such teeth are suggested to pull out the cribellate fibres, although not all cribellate spiders do have teeth on their calamistra (e.g. Uloboridae) and the calamistra itself has proved not to be necessary for fibre extraction [7,19]. Furthermore, contact between cribellate teeth and cribellate thread during capture thread production should be inhibited, as this study has shown contact between fibres and calamistrum occurs at the surface-like area. Hence it is unlikely that the teeth have a function during the cribellate thread production.

However, *Deinopis* sp. showed a conspicuous elongated posterior leg movement in the last combing cycle, after which fibres were attached to the calamistrum. Such an attachment at the fourth leg prior to radial attachment has already been earlier described [18,34]. As *U. plumipes* and *Z. geniculata* adapted their opisthosoma position during each single combing movement to prevent changed contact, this last elongated leg movement of *Deinopis*sp. has to have an impact on the contact by lowering *α*. This in turn should change the contact from the surface-like area near the basis of the calamistrum to the thinner tips of the setae. Hence the occurring contact area is comparable to the one observed in Uloboridae (figure 3*g*–*i*). However, in contrast to the Uloborids’ calamistra, the tips of the calamistrum of *D. subrufa*bore teeth (figure 3*j*). Hence we suggest these cribellate teeth come in contact with the cribellate fibres after the last elongated leg movement and therefore help in picking up the thread and handling it independently of the spinnerets.

Our hypothesis is supported comparing the occurrence of such teeth in cribellate spiders: Uloboridae like *U. plumipes* and *Z. geniculata* do not have cribellate teeth. However, they do not need to move the thread independently of their spinnerets, as they move their whole body due to their web building behaviour to the attachment site [16,31–33]. For other spider families like Deinopidae, Amaurobiidae, Eresidae, Filistatidae, Hypochilidae or Zoropsidae, the occurrence of teeth is described [18,35–37]. These spiders have in common that they do not constantly (or at all) move during thread production [17,23,38,39]. Consequently, they have to move the produced thread independently of their spinnerets to its final destination. Although we can provide here a plausible hypothesis for the purpose of the cribellate teeth, real evidence is needed to verify their function.

## 5. Conclusion

In this study, we demonstrate that the cribellate thread production model established with *U. plumipes* is also transferable to other species. Although both families examined in this study (Uloboridae and Deinopidae) are closely related to each other, they show striking morphological different calamistra. These differences nevertheless do not influence the preserved cribellate capture thread production process and furthermore have no function in processing the nano fibres differently. The morphological differences seem to be adaptations owing to differences in their web building behaviour. These lead to a shifted contact area on the calamistrum and furthermore different needs for handling the capture thread.
